# Uncovering spatial and social gaps in rural mobility via mobile phone big data

**DOI:** 10.1038/s41598-023-33123-0

**Published:** 2023-04-20

**Authors:** Zhengying Liu, Pengjun Zhao, Qiyang Liu, Zhangyuan He, Tingting Kang

**Affiliations:** 1 School of Urban Planning and Design, Peking University Shenzhen Graduate School, Shenzhen, 518055 Guangdong China; 2 College of Urban and Environmental Sciences of Peking University, Beijing, China; 3 Key Laboratory of Earth Surface System and Human-Erath Relations of Ministry of Natural Resources of China, Shenzhen, China

**Keywords:** Sustainability, Socioeconomic scenarios

## Abstract

Rural mobility inequality is an important aspect of inequality-focused Sustainable Development Goals. To reduce inequality and promote global sustainable development, more insight is needed into human mobility patterns in rural areas. However, studies on rural human mobility are scarce, limiting our understanding of the spatial and social gaps in rural human mobility and our ability to design policies for social equality and global sustainable development. This study, therefore, explores human mobility patterns in rural China using mobile phone data. Mapping the relative frequency of short-distance trips across rural towns, we observed that geographically peripheral populations tend to have a low percentage of short-distance flows. We further revealed social gaps in mobility by fitting statistical models: as travel distances increased, human movements declined more rapidly among vulnerable groups, including children, older people, women, and low-income people. In addition, we found that people living with low street density, or in rural towns in peripheral cities with long distances to city borders, are more likely to have low intercity movement. Our results show that children, older adults, women, low-income individuals, and geographically peripheral populations in rural areas are mobility-disadvantaged, providing insights for policymakers and rural planners for achieving social equality by targeting the right groups.

## Introduction

Rural areas remain the sites of many and varied social problems. For example, extreme poverty is predominantly a rural phenomenon^1^. Improving rural mobility is crucial to the global objective of reducing poverty and promoting rural development^2,3^. Human mobility in rural areas has gained the attention of international organizations and governments in many countries. For instance, the World Economic Forum^4^ indicated that reforming rural mobility will energize rural economies. In South Africa, the Rural Transport Strategy developed by the Department of Transport^5^ emphasized the important role of improving rural mobility in reducing poverty. In China, policy attention has turned to the goal of achieving common prosperity. However, rapid urbanization in China has caused many problems, such as imbalances in rural and urban development^6^, making mobility a source of novel inequalities that further widen the gap between urban and rural areas^7^. To the end of achieving widespread prosperity, promoting human mobility and reducing mobility inequalities have become critical policy priorities of the Chinese government^8,9^.

Mobility inequality exists not only between urban and rural areas but also within rural areas. As a core dimension of Sustainable Development Goals (SDGs) related to inequality, mobility inequality should be taken into account by local policies aimed at reducing inequality and promoting global sustainable development^10,11^. To make more informed and effective policy decisions, more insight is needed into human mobility patterns in rural areas. Improved understanding of human mobility patterns also has practical implications for traffic forecasting^12^, epidemic control^13^, emergency management^14^, etc. To capture and undestand human mobility patterns, researchers have used trip distance distribution as a significant statistical property. For example, Yao and Lin^15^ examined the distance distribution of human mobility by single vehicle, finding that mobility distance by taxi follows a power-law distribution. Tang et al.^16^ used trip distance to characterize human mobility by urban taxi, finding that occupied taxi trip distance distribution follows a combined pattern, i.e., one part follows a power-law distribution, and the other follows a truncated power-law distribution. Hipp and Boessen^17^ estimated the distance decay function over both intra- and intermetropolitan moves, and found that a log-linear representation appeares to capture this distance decay function.

However, human mobility research to date has largely focused on urban areas, with limited insights available on human mobility in rural areas. Human movement patterns in rural areas may differ significantly from those in urban ones, influenced by such factors as geographical barriers and transport availability. For example, one study compared the distribution of human travel in urbanized and rural societies, finding that these follow different statistical distributions^18^. When used to predict human movement, the standard gravity model performs well when applied to intercity movement, but poorly when applied to rural activity^19^. Therefore, the existing literature on human mobility might not be applicable to rural mobility specifically; there remains a need to better understand the characteristics of human mobility specific to rural areas.

Moreover, few studies have analyzed the socio-demographic differences in human mobility patterns in rural areas. It has been shown that human movement patterns in urban areas vary across socio-demographic characteristics. For example, Kraemer et al.^20^ examined global human mobility patterns and found that human movements decline faster as a function of distance in low-income countries than in high-income countries. Luo et al.^21^ compared the differences in mobility characteristics of Twitter users in Chicago across different demographic groups, finding that race/ethnicity and age affect urban mobility patterns. However, it is unclear whether these socio-demographic differences identified in urban mobility patterns also exist in rural environments. An enhanced understanding of the socio-demographic variations in human mobility patterns is relevant to various real-world applications, such as reducing social inequality, controlling the spread of infectious diseases, and improving transportation systems’ performance. Therefore, it is necessary to examine how human mobility patterns in rural areas vary across socio-demographics.

Human mobility studies conventionally rely on census data and traditional surveys of travel journals as primary data sources^22^. The process of a conventional survey is generally long and expensive, thus is often difficult to conduct in rural areas. The lack of research on human mobility in rural areas may be attributed to scarce data availability. However, advances in Web and mobile technologies have introduced a set of cost-effective and reliable data sources that can provide large-scale, granular spatiotemporal information on human behaviors, even in rural areas. Examples of these data sources include mobile phone signaling data^23^, vehicle GPS trajectories^15^, and transit smart cards^24^. Researchers have conducted numerous studies on urban human mobility patterns at the intra- and intercity levels that leverage these data sources. However, studies on human mobility in rural areas and at the regional scale are still limited in number.

To address the above-mentioned research gaps, this study used mobile phone signaling data to explore the characteristics of human mobility in rural areas and uncover the spatial and social gaps in rural mobility. The Greater Bay Area (GBA) was selected as a case study because it is representative of many different rural Chinese environments. Moreover, as one of the most vibrant economic agglomerations in China, the GBA hosts an increasing flow of people across rural areas located near different cities, making it a meaningful site within which to examine regional-level characteristics of human mobility. In addition, the GBA (excluding Hongkong and Macao) is located in Guangdong Province, where mobile cellular subscription rates in 2022 were 131.4 per 100 people, the highest in China^25^. This high rate of mobile phone usage and correspondingly large amount of signal data makes it possible to track rural human mobility patterns in the GBA region with confidence.

The remainder of this paper is organized as follows. The next section introduces the study data and research methods. The third section evaluates the rural human mobility patterns in terms of spatial distribution of short-distance trips, socio-demographic variations in the distribution of long-distance trips, and intercity movement patterns. The fourth section discusses rural human mobility patterns. The paper concludes with the policy implications of our analyses and a discussion of the study’s contributions and further applications.

## Results

### The geography of short-distance trips

In general terms, mobility is defined as the ability to move freely and easily, where “freely” and “easily” in fact represent different variables. When residents of two areas travel a relatively short distance (e.g., 10 km), the ease of movement tends to be similar; in this condition, then, the variance in mobility among residents is in their respective abilities of free movement. Consider two rural towns: the first with good-quality infrastructure nearby, the second with poor-quality infrastructure. Residents in rural towns with better infrastructure have the freedom to travel both short and long distances to access various services. By contrast, residents in rural towns with poor infrastructure must travel longer distances to access better services, due to lower freedom of movement. It is therefore likely that the higher frequency of short-distance trips observed in the first town is due to the higher level of free movement it affords its residents. In other words, relative frequency of short-distance trips is a potential indicator of residents’ relative freedom of mobility. When traveling longer distances, however, ability to move easily tends to be the decisive factor. Thus, ability to move easily might be a better indicator than ability to move freely when accounting for variations in mobility among residents.

**Figure 1:**
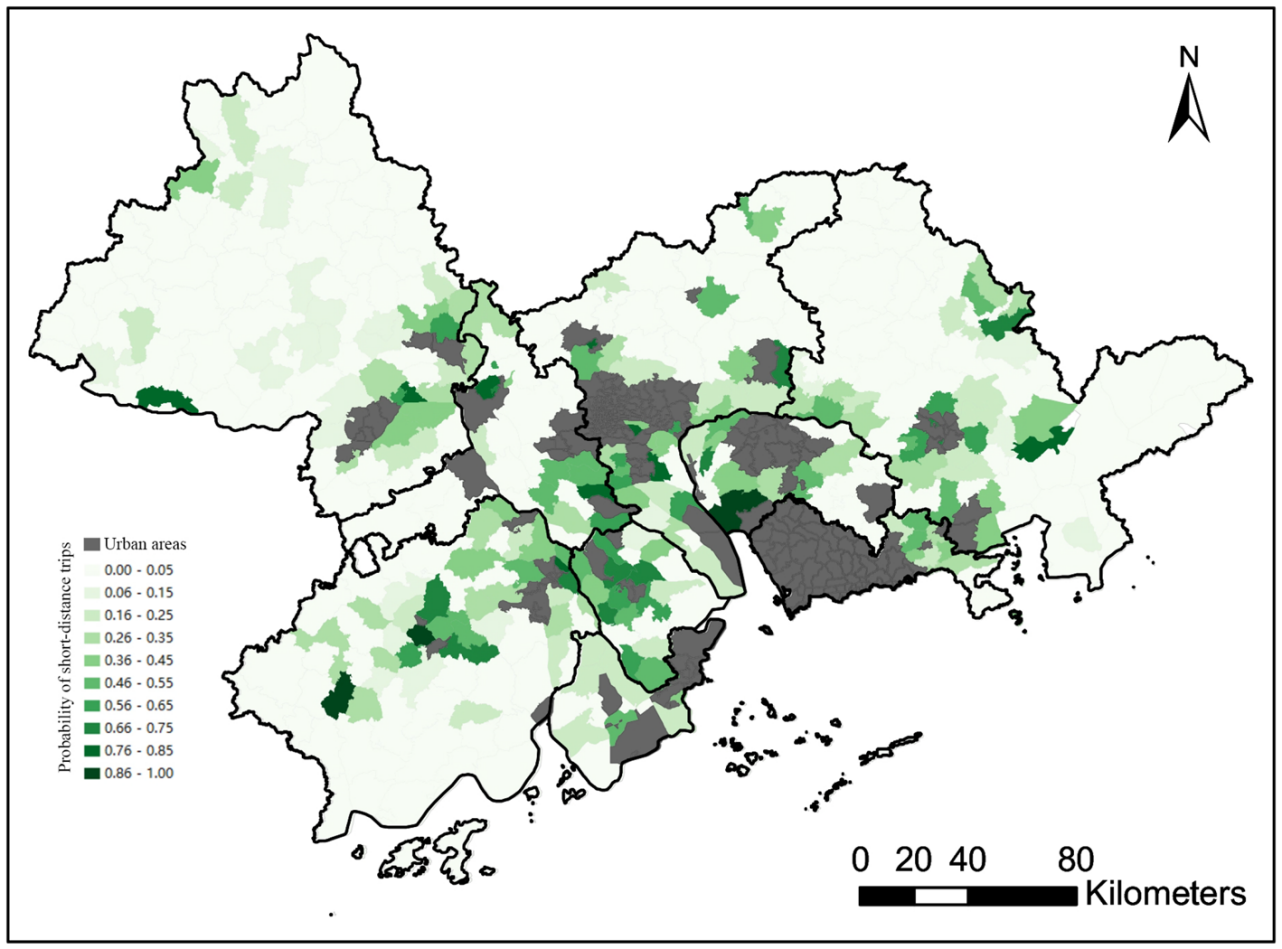
Spatial variation in the relative frequency of short-distance trips across rural towns.

**Figure 2:**
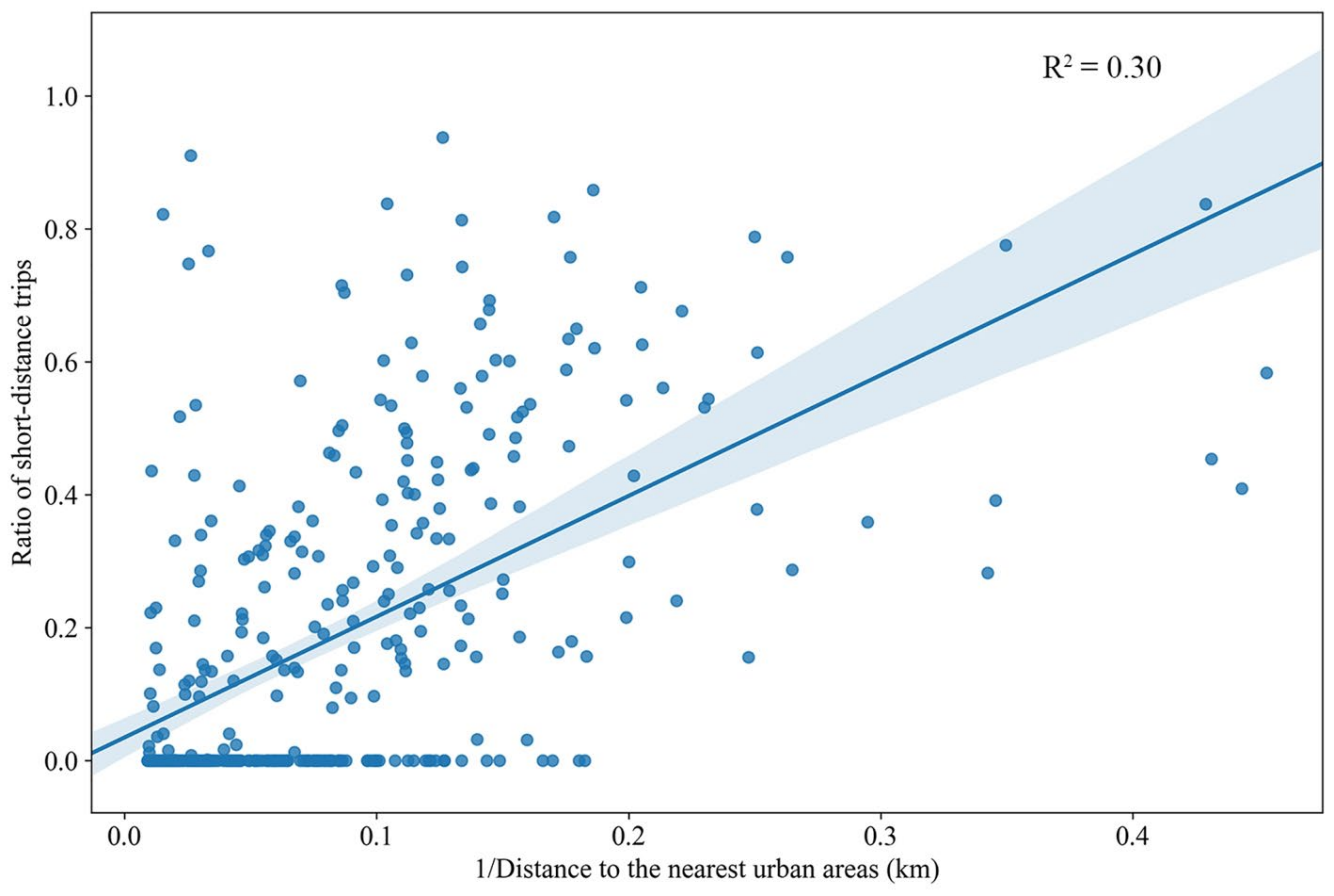
Scatter plot with the ratio of short-distance trips as a function of the inverse of the distance to the nearest urban areas.

In this study, we first characterized human mobility by looking at the relative frequency of short-distance trips for each rural town. Figure 1 shows the spatial variation in the relative frequency of short-distance (within 10 km) trips across rural towns. Darker colors indicate a higher frequency of traveling within 10 km. The majority of rural towns located at the geographical center of the GBA have a large percentage of flows within 10 km, while rural towns at the fringes of the GBA tend to have a low percentage of short-distance trips. This may reflect the fact that rural residents living at the fringes of the region tend to need to travel long distances to access quality health care, shopping, employment opportunities, etc. Moreover, compared to rural towns located near urban areas, those farther away generally have long-distance flows (Fig. 2), indicating that the movements of residents living near urban areas tend to be more spatially localized than those living farther away.

### Variation of human mobility across socio-demographic groups

#### General description

**Figure 3:**
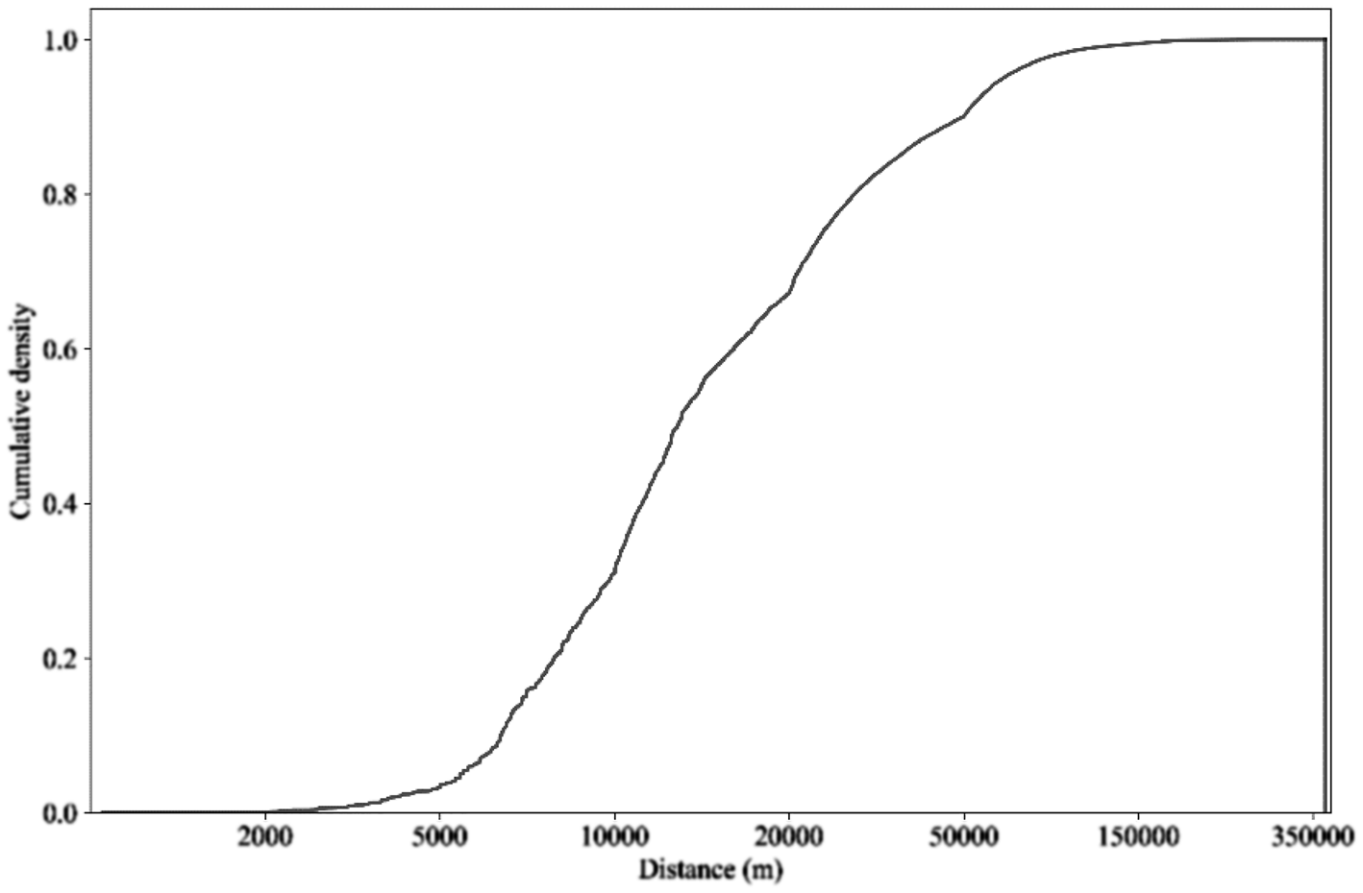
Distribution of travel distances of all individuals.

Another key aspect in determining human mobility is the distribution of distances traveled. The mean regional trip distance was 22.8 km (median 13.6 km), including both intracity and intercity movements, which is heavily influenced by long-distance trips with > 15,000,000 movements > 100 km, and the maximum distance recorded was 398.5 km. Trips with distances over 50 km accounted for 10% of all recorded flows (Fig. 3).

**Figure 4:**
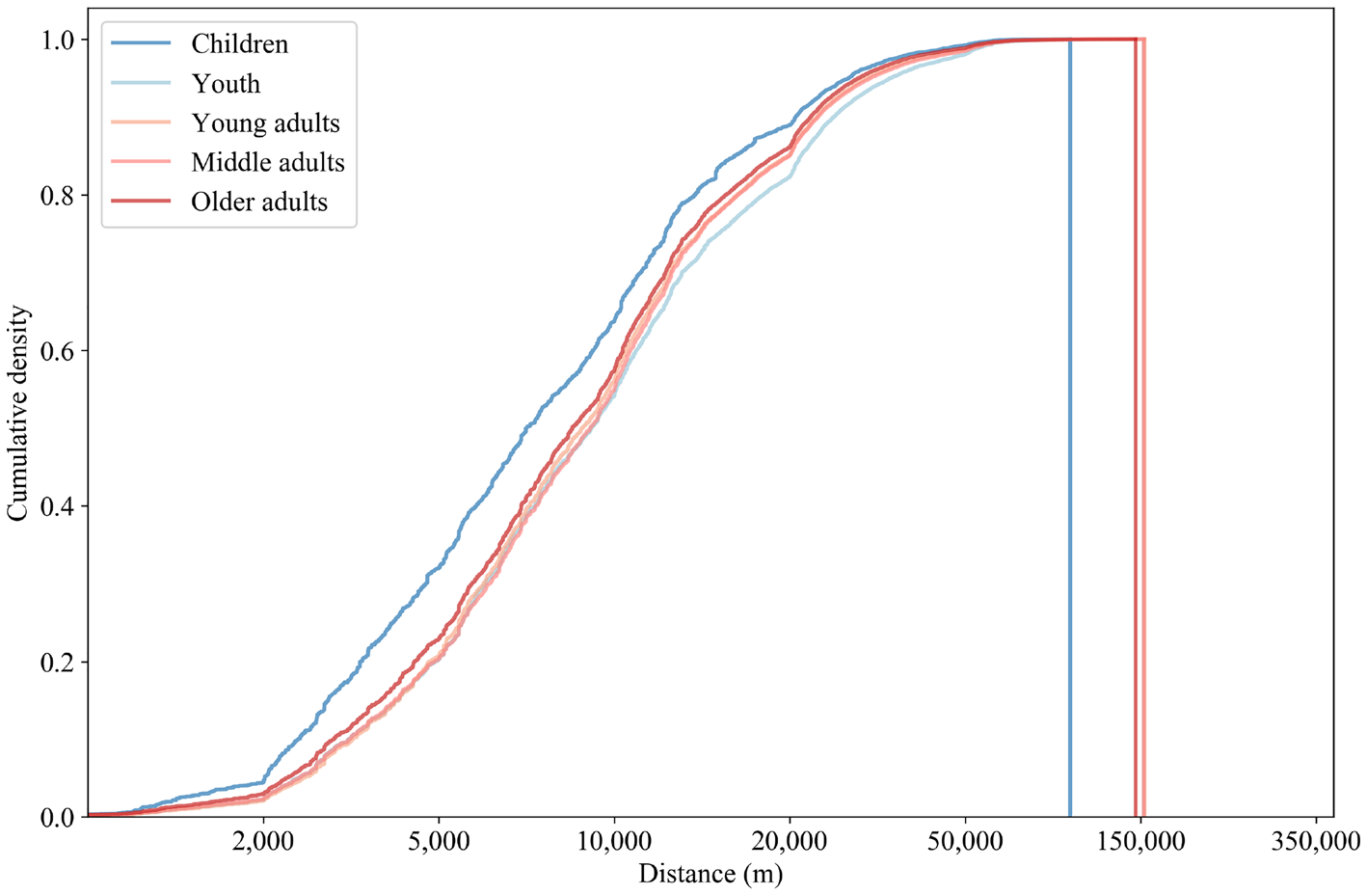
Distribution of trip distances across age groups.

**Figure 5:**
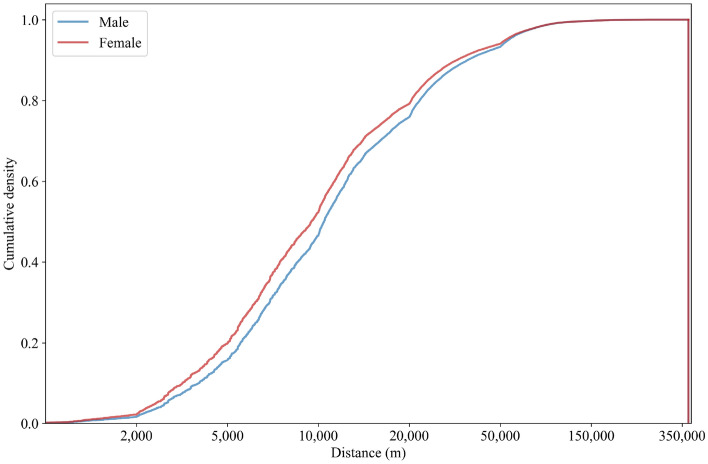
Distribution of trip distances across genders.

**Figure 6:**
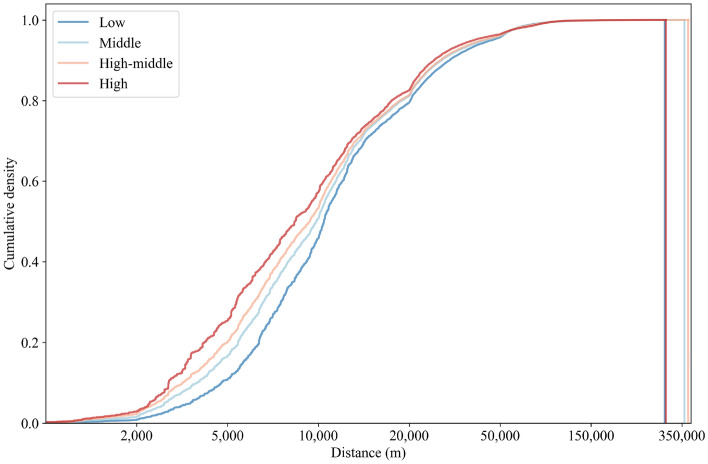
Distribution of trip distances across income groups.

The distributions of travel distances vary by many socio-demographic factors, including age, gender, and income (Figs. 4, 5, 6). Table 1 shows the distribution of the sample by age, gender and income. The sample is basically representative of the socio-demographic structure of the GBA in terms of gender ratio and income group distributions. Although the distribution of mobile phone users by age does not closely represent the real age distribution in the GBA, this age group breakdown for mobile phone users matches well with the smartphone users survey in China conducted by the Mobile Ecosystem Forum^26^. Therefore, we believe that our dataset is representative of the most mobile phone users in terms of age group distribution.

**Table 1:** Descriptive characteristics of the sample (rescaled).

Number of total residents in the GBA (million)	Number of users (million) (unidentified age)	Number of users (million) (identified age)					
		Total	Children	Youth	Young adults	Middle adults	Older adults
78.23	19.03	59.20	0.01	3.14	38.86	14.78	2.41
100%	24.33%	75.67%	0.01%	4.01%	49.67%	18.89%	3.08%

	Number of users (million) (unidentified gender)	Number of users (million) (identified gender)					
		Total	Male	Female			
	16.20	62.03	33.32	28.71			
	20.71%	79.29%	42.59%	36.70%			

	Number of users (million) (unidentified income)	Number of users (million) (identified income)					
		Total	Low	Middle	High-middle	High	
	36.06	42.17	10.59	13.87	16.65	1.06	
	46.09%	53.91%	13.54%	17.73%	21.28%	1.36%	

The distribution of trip distances for different age groups is shown in Fig. 4. The median distance traveled by children versus youth varies by a factor of 1.25, where 50% of movements occur at 7.5 km versus 9.4 km, respectively. The median distance traveled by older adults is the next-shortest. Short-distance flows among adults are similar in frequency. In youth, the frequency of long-distance movements is larger (12% of movements occur above 25 km) compared with children, young adults, middle adults, and older adults, with 7%, 10%, 10%, and 9% (respectively) of movements occurring above 25 km.

The distributions of trip distances by gender are shown in Fig. 5. The median trip distance of males (10.6 km) is larger than that of females (9.6 km). Moreover, the distribution of both genders’ travel distances can be decomposed into three parts: the first part is 0–2 km, the second is 2–50 km, and the third is 50–350 km. Specifically, the male and female travel distance distributions are very close in the first and third parts; in distances from 2 to 25 km, however, the probability density for females is higher. This means that travel by women is more localized than travel by men.

The distributions of trip distances for different income groups are shown in Fig. 6. The highest median trip distance (10.5 km) is in the low-income group, followed by the middle-income group (9.8 km). The lowest median trip distance is in the high-income group (9.1 km). This may imply that high-income groups in rural areas travel shorter distances than low-income groups to access needed goods and services. By examining the spatial distribution of income across rural towns, we found that most high-income groups live in rural towns close to urban areas. We therefore deduced that the shorter median trip distances in high-income groups may owe to the fact that high-income groups live closer to urban areas and have better infrastructure nearby; thus, they do not need to travel long distances to access various goods and services. Moreover, we found that the frequency of short-distance flows in high-income groups is higher than that in other income groups. This, too, may reflect the fact that high-income groups typically live in areas wherein most of their daily needs can be met within a short distance.

#### Fitting power law

To understand the robustness of our data, we fit simple statistical models to the GBA’s rural human movement patterns. Generally, the frequency of human movement decreases with travel distance^27,28^ and follows a power law. However, these analyses of human movements have been critiqued as limited to small sample sizes^29,30^. To understand the variability of human movements across socio-demographics, and to formalize the variations in human movements, we used the truncated power law with an exponential cutoff in the form of Eq. (1).

**Figure 7:**
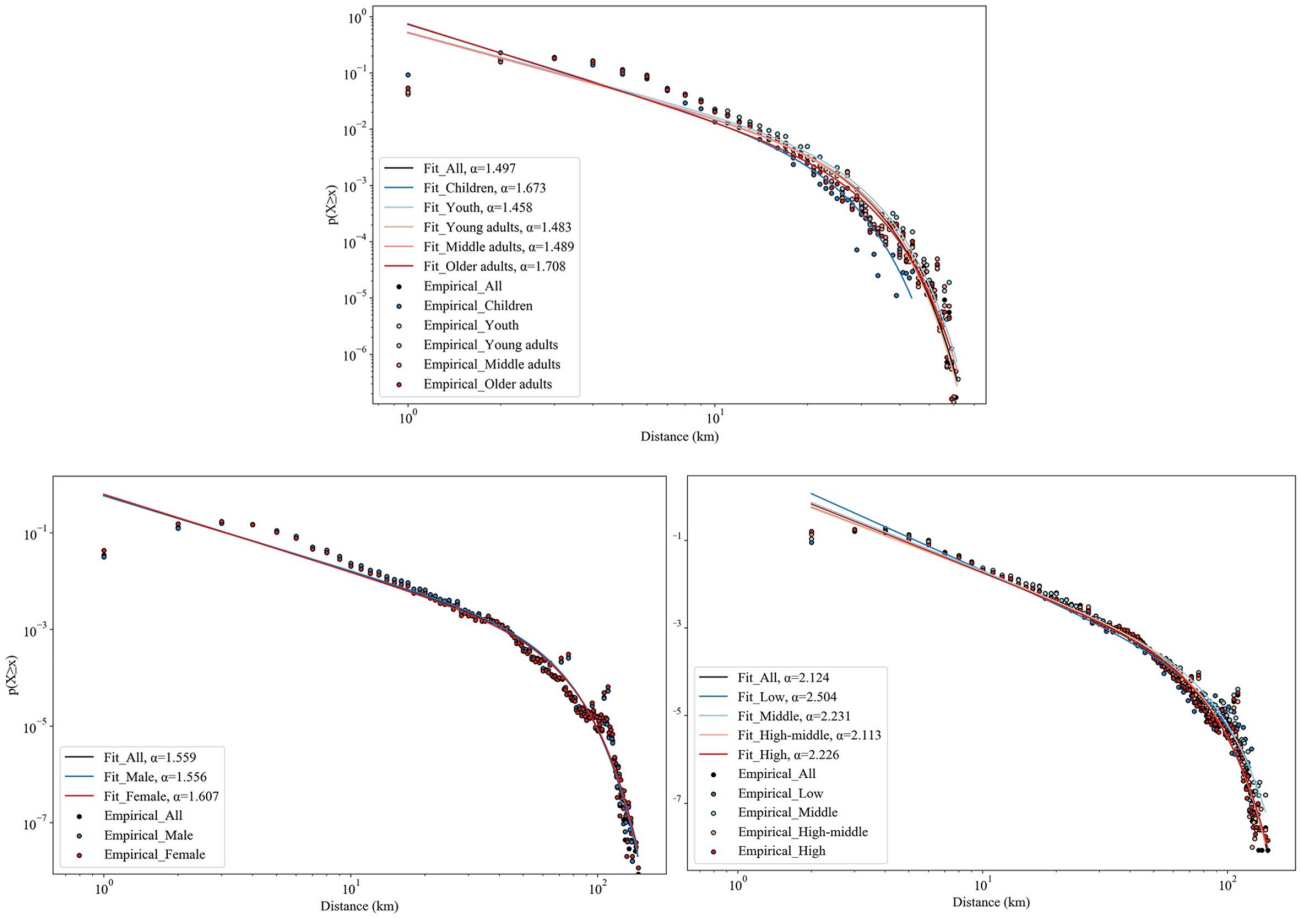
Values of scaling coefficient (α) of fitted truncated power-law distributions for different groups.

We then estimated the power-law exponent in different socio-demographic groups (Fig. 7). We found that the distribution of the scaling parameters of five age groups has a “U” shape, with a higher scaling parameter in children (*α* = 1.673), older adults (*α* = 1.708) and middle adults (*α* = 1.489) than in youth (*α* = 1.458) and young adults (*α* = 1.483). Moreover, we found that human movements decline faster as a function of distance in females than in males (*α* = 1.607 and 1.556 for females and males, respectively). In addition, we observed that people with low income have a higher scaling parameter (*α* = 2.504) than all other income groups (*α* = 2.231, 2.113, and 2.226 for people with middle, high-middle, and high income, respectively). These results illustrate that the human movements of vulnerable groups, including children, older people, females, and low-income people, are more susceptible to distance.

### The geography of intercity human movements

**Figure 8:**
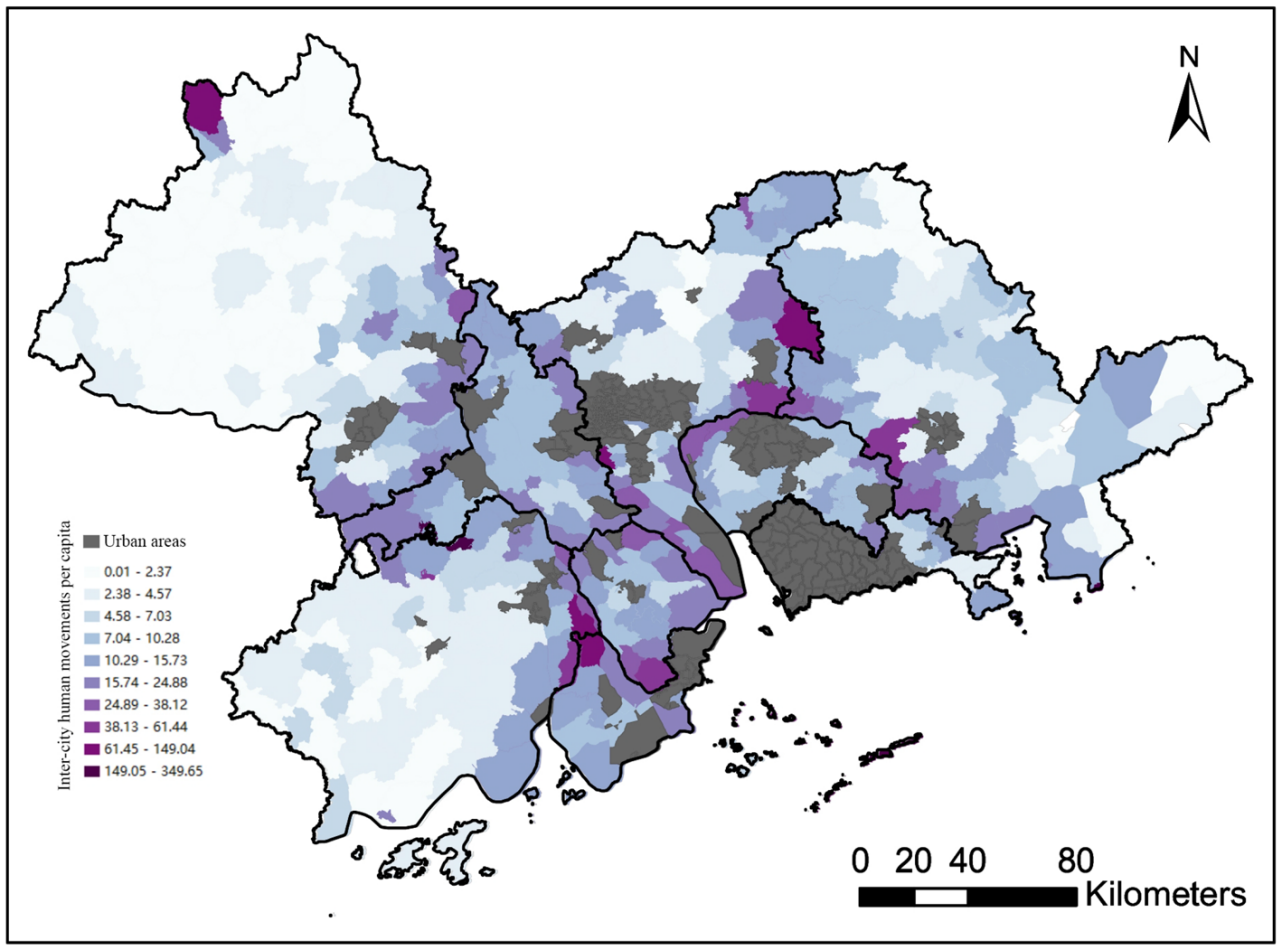
Spatial variation in intercity human movements per capita for rural towns.

As the third indicator of human mobility, we used intercity movements per capita at the town level. Since human movements are considered a powerful enabler of rural development^31,32^, we argue that rural towns with higher intercity movements per capita are more likely to have better development potential and therefore achieve rural revitalization. In the GBA, there are an average of 12.62 intercity movements per capita per month. Figure 8 shows that most rural towns located at the convergence of cities have larger intercity movements. In addition, compared to the rural towns near cities in the central GBA (i.e., Foshan, Guangzhou, Zhongshan, Dongguan), most rural towns near cities in peripheral GBA areas (i.e., Zhaoqing and Jiangmen) have lower intercity movements.

**Figure 9:**
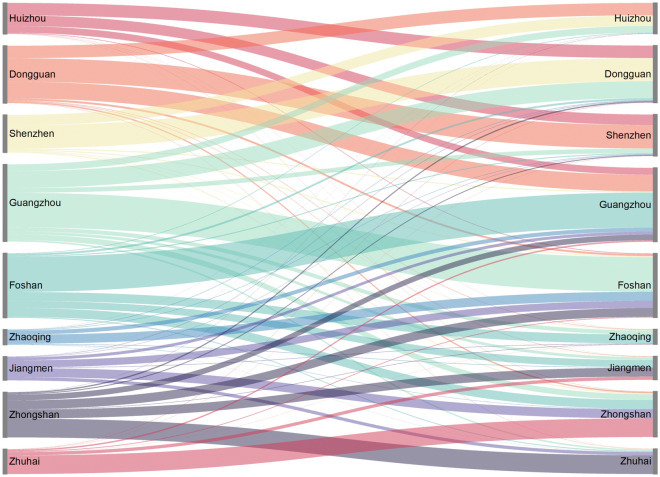
Recorded bilateral intercity movements for the city pairs in the GBA.

We further differentiated city pairs with a high frequency of intercity movements, aggregated from flows among rural towns located near different cities. Figure 9 shows that people travel predominantly between Dongguan–Shenzhen, Guangzhou–Foshan, and Zhongshan–Zhuhai. Although Zhaoqing, Jiangmen, and Zhongshan are also adjacent to Foshan, there are significantly fewer connections between these cities and Foshan than those between Guangzhou–Foshan. There are also relatively fewer connections between Guangzhou–Dongguan and Huizhou–Dongguan than between Shenzhen–Dongguan, despite their comparable proximity. The strong connections between these three city pairs (i.e., Dongguan–Shenzhen, Guangzhou–Foshan, and Zhongshan–Zhuhai) may be attributed to the integrated development policy of these cities.

This study adopted a linear mixed model to analyze the factors influencing intercity movements. This model assumes that the dependent variable is normally distributed. Since the dependent variable, i.e., the intercity movements per capita, was not normally distributed, we transformed it to normally distributed by applying the Box-Cox transformation. Distance to city borders, street density, and regional policy were introduced to the regression model. The effects of regional policy on intercity movements were represented by one dummy variable. The reference category was the origin city without integrated development policy.

The estimation results are presented in Table 2. A Marginal R-squared of 0.428 and a conditional R-squared of 0.447 indicated the model’s high goodness of fit of the model. The model shows that there is a significant reciprocal relationship between distance to city borders and intercity movements. The effect of street density of local towns on intercity movements is also significant. Regarding the effects of regional policy on intercity movements, the results indicate that people in rural areas in cities with integrated development policy are likely to have higher intercity movements, compared to those in cities without integrated development policy. Moreover, there are random effects for regional policies, meaning that the effects of regional policies on intercity movements vary among cities.

**Table 2:** Linear mixed model estimates for intercity human movements per capita.

Variables	Coefficient	*P* value
Fixed effects		
1/distance to city borders (km)	3.600	< 0.001
Street density (km/km^2^)	0.305	0.005
Origin city with integrated development policy		
Yes	0.367	0.047
Intercept	1.112	< 0.001
Random effects		
Regional policy (variance)	0.070	
Goodness of fit		
Marginal *R*-squared	0.428	
Conditional *R*-squared	0.447	

## Discussion

A better understanding of rural human mobility patterns is crucial to reducing inequality and promoting global sustainable development. It also carries practical implications for, for instance, traffic forecasting^12^, epidemic control^13^, emergency management^14^. However, there have been few evaluations of the characteristics of human mobility in rural areas. Based on human movements as estimated from mobile phone data, we explored the spatial and socio-demographic characteristics of human mobility in rural areas.

Our findings suggest that compared to rural towns that are near urban areas, those far away from urban areas generally have long-distance flows. This could imply that the farther rural towns are from urban areas, the more likely they are to have long-distance flows. This may reflect inadequacies in the healthcare facilities, shopping facilities, employment opportunities, etc. available in rural areas, and the subsequent need to travel long distances to obtain these things. This finding is in line with Zhao and Cao^24^ who found that people living in disadvantaged areas, i.e., with low job accessibility and poor transport services, tend to endure traveling long distances to access jobs and public services.

We also found that the distance distribution of rural human mobility follows a truncated power law. This is consistent with previous studies showing that the distance distribution of traveling to places outside the immediate vicinity follows a truncated power law^30,33^. Moreover, we found that rural human mobility is highly heterogeneous across socio-demographic groups. Specifically, human movements decline more rapidly among vulnerable groups, including children, older people, females, and low-income people, as travel distance increases. This finding is in line with Luo et al.^21^ who found that women and older adults travel shorter distances than men and young people, and Kraemer et al.^20^ who concluded that human movements decline more rapidly in low-income settings. Overall, this research is consistent with previous research on human mobility in demonstrating that human mobility varies across socio-demographic groups, whether in rural or urban areas.

Our analysis of intercity human movements revealed that they are highest among cities with integrated development policy and towns with shorter distances to city borders or high street density. Although the intercity integration development policy mainly promotes connections between urban areas, our findings suggest that the connections between rural areas may also benefit from such policies. Moreover, we found that intercity movements increase markedly with proximity to city borders. This means that the city-adjacent areas may have a high demand for intercity bus services; thus, corresponding transport infrastructure should be provided. This finding is consistent to some degree with Kraemer et al.^20^ who found that there is a positive relationship between the frequency of international trips and the proximity to an international border. In addition, we found that there is a positive relationship between street density and intercity movements. This indicates that rural transport policies, such as investing more in rural roads and improving rural road networks, may be effective in promoting intercity movements of populations in rural towns.

The present study has some limitations. First, we used data gathered over a one-month period only. This is enough to reveal relevant patterns of human mobility in the GBA’s rural areas; however, we may need data from a much longer period to demonstrate such patterns definitively. Second, there is potential bias in the mobile phone datasets. Although the datasets capture a large proportion of residents in the GBA, certain demographic tiers, e.g., older adults, tend to use mobile phones less frequently and thus might be underrepresented. While we found that movements varied substantially among older adults and younger age groups, we anticipate that these differences would be even greater when GBA’s total rural populations are considered. Finally, due to privacy concerns, mobile phone data are available only in the aggregated form. If this data were made available in disaggregated form, we could develop additional mobility metrics, such as the Shannon mobility entropy^34^ and the radius of gyration^29^, to examine more aspects of rural mobility.

This study contributes to our understanding of the characteristics of human mobility in rural areas. Overall, the spatial distribution of the relative frequency of short-distance trips across rural towns demonstrates the difference in rural human movement patterns across space. Moreover, we found that universal power laws govern rural human mobility but are highly heterogeneous across socio-demographic groups. We further found that intercity human movements are highest in (a) cities with integrated development policy and (b) towns with short distances to city borders or high street density.

The findings from this study have many important practical implications. First, long-distance travel is a significant factor in transport inequity. This study demonstrates that most rural towns located on the fringes of the region have a high percentage of long-distance flows, suggesting to policymakers that interventions to reduce transport inequity are urgently needed in these areas. Second, we found that rural human movements vary across socio-demographic groups. Specifically, human movements of vulnerable groups, including children, older people, women, and low-income people, are more susceptible to travel distance. In other words, increased travel distances can rapidly result in a reduction of the mobility of disadvantaged people as compared with advantaged people. This insight can help decision-makers control the spread of infectious diseases more effectively by targeting vulnerable population groups. Moreover, from the perspective of reducing transport inequity, these disadvantaged groups should be paid more attention. Third, integrated development policies should be encouraged to promote intercity human movements and thus drive the economic and social development of rural towns near the city, since intercity human movements are predominantly between cities that have enacted such policies. Finally, given the positive relationship between street density and intercity human movements, investing further in rural roads and improving street density could help reduce human mobility inequality in terms of intercity movements.

This study makes some important contributions to the literature regarding human mobility. First, it adds to the limited empirical evidence on the spatial and socio-demographic characteristics of human mobility in rural areas and represents one of the first attempts to examine human mobility in rural areas at the inter-town level and the regional scale. Second, it demonstrates that universal power laws govern rural human mobility but are highly heterogeneous across socio-demographic groups. Finally, it reveals the importance of integrated development policies, distance to city borders, and street density as drivers of intercity human movements.

## Methods

### Data

Raw mobile phone data from the GBA (from November 1, 2019 to November 30, 2019) were provided by China Unicom, one of the three largest telecom companies in China. The original data included the time the user communicated with a base station and the base station’s coordinates. To protect personal information privacy, the mobile phone numbers of subscribers were anonymized by the mobile phone operator inside their premises and anonymized mobile signaling data were never transferred outside of the operator’s system. Users’ daily trajectories were extracted from the original data and collected in a background computation platform: an algorithm within the platform then collected user behaviors within specific spatial units, yielding the number of visits. Moreover, mobility data used in this study were aggregated according to time, space, and user attributes. This means that the analysis never singled out identifiable individuals. To exclude transfer passengers, single visits were defined as town-level units lasting for ≥ 3 h. Given the fact that people who are present in a town during the study period cover permanent and temporary residents, we excluded those who temporarily stay so that we can capture the real characteristics of local people’s mobility. To exclude temporary residents, we only selected users who stay at home for more than 20 days in a month. The data cell where a user had the longest stop during the night was defined as his or her home. If some of the permanent residents just left the GBA during the study period, the dataset would not include them. We believe such cases account for a tiny faction and thus it will not influence the representativeness of the results. Since only a portion of GBA’s total population uses China Unicom’s mobile phone network, the data cannot adequately represent the actual population flow in the GBA. Therefore, accounting for China Unicom’s market share in each town, we rescaled our results to describe the actual population in each town.

Due to China’s regulations on user information, mobile phone users’ socio-demographics (e.g., age, gender) were available. Since September 1, 2015, all mobile phone users have had to provide their personal information (e.g., an identifying card number) to apply for SIM cards^35^; from this information, their age and gender are detectable. According to their age, the users were classified into five age groups: children (≤ 12 years), youth (13–18 years), young adults (19–39 years), middle adults (40–59 years) and older adults (≥ 60 years). Income levels were deduced from users’ activity space, geographic exposure, mobile phone price, and the average market price of a house in their residence district. Research shows that activity space, geographic exposure, mobile phone price, and housing price are closely correlated with user income^36–38^. Based on analysis of the above information, the users were classified into four groups: high-income group, high-middle income group, middle-income group, and low-income group.

Road network data used to calculate street density within a town administrative area were obtained from the Open Street Map in 2020.

### Identification of rural towns

The identification of rural towns is as follows: First, according to the Rural–Urban Continuum Codes developed by the National Bureau of Statistics of China^39^, we classified all town-level units into three types, i.e., urban areas, rural areas, and others. The Rural–Urban Continuum Codes (111, 112, 121, 122, 210, 220) delineate all village/community-level units such as downtown, town center district, village, etc. A town will be designated as urban if all village/community-level units within the town are coded 111, or as rural if all village/community-level units within the town are coded 220. In all other cases, a town will be designated as others. Second, we calculated the areas of the built-up areas based on land use/land cover maps of the GBA. All towns were classified into five groups using a natural breaks method. For towns designated as others during the first step, they will be designated as urban if they are in the first group, as urban–rural fringe if they are in the second or third group, or as rural if they are in the fourth or fifth group. Finally, all town-level units in the GBA were classified into three types including 310 rural areas, 72 urban–rural fringe areas, and 262 urban areas. We defined towns that are not located in urban areas as rural towns.

### Analysis

#### General description of human movement

Rural human movement patterns may vary across geographies. Therefore, we used spatial analysis and summary statistics to compare human movement patterns between rural towns. First, we computed the percentage of short-distance trips in each town. Since most of the daily needs of residents in rural areas are met within a 10 km radius from the point of residence^40^, we defined “short-distance” as < 10 km. The percentage of short-distance trips was calculated by dividing the total number of movements within the threshold travel distance by the total movements. To obtain insight into how far people move on average, for each rural town, we calculated the percentage of movements from the origin point, from 0 to the maximum km, in 1 km increments (Figs. 3, 4, 5). To compare how distance kernels differ by socio-demographic group, we made pairwise comparisons between the distributions at 1 km increments, from 0 to the maximum km.

#### Fitting the statistical power-law model

Research on human mobility has shown that there is a negative relationship between the travel distance and the probability of traveling between sites, and the probability of traveling follows a truncated power law. However, the scaling parameter may differ across geographies^30,33^. To examine the distance distribution of human movement in rural areas, we fit a truncated power law with the form of Eq. (1).

1 $$p\left(x\right)\propto {x}^{-\alpha }{e}^{-\lambda x},$$

where *α* is a constant parameter of the distribution (known as the scaling parameter or exponent), *x* is the travel distance (*x* > *x*_min_ > 0), and *λ* is the parameter of the exponential distribution. *x*_min_ represents the shortest distance above which the scaling relationship of the power law begins. The scaling parameter *α* must be estimated before finding the optimal values of *x*_min_. The methods of Clauset et al.^39^ find *x*_min_ by creating a power law fit beginning from each unique value in the dataset, then choosing the one that leads to the minimal Kolmogorov–Smirnov distance between the data and the fit. For any given value of *x*_min_, we estimated the scaling parameter using maximum-likelihood estimation. The goodness of fit for truncated power law distribution was considered by comparison to the fit of other distributions (e.g., power law distribution, exponential distribution, and lognormal distribution). We used standard Python packages to produce results for this analysis.

#### Characteristics of intercity human movements

To characterize intercity human movements, we first calculated and mapped the intercity movements per capita at the town level. Specifically, for each town, we calculated the ratio of the intercity flows of populations to the total population size. We then aggregated the town level intercity movements to the city level and depicted the flow relationship between cities using a chord diagram. Finally, we estimated the effects of distance to city borders, street density, and regional policy on intercity human movements using a linear mixed effects model. Linear mixed effects models include random intercepts models, random slopes models, and random intercepts and slopes models. We applied a random slopes model because rural towns near the same cities share the same regional policies, implying that the impacts of regional policies on intercity movements between these towns can be correlated.

The structure of the proposed random slopes model is presented in Eq. (2)

2 $${Y}_{ij}= {\beta }_{0}+{\beta }_{1}{X}_{ij1}+{\beta }_{2}{X}_{ij2}+ {b}_{i1}{Z}_{ij1}+ {\varepsilon }_{ij},$$

where ${Y}_{ij}$ is the intercity movements per capita for the *j*th rural town in city *i*, and ${X}_{ij1}$, ${X}_{ij2}$, and ${Z}_{ij1}$ are the independent variables, i.e., distance to city borders, street density, and regional policy, respectively. ${\beta }_{0}$, ${\beta }_{1}$, and ${\beta }_{2}$ are the fixed effects parameters shared by all cities, and ${b}_{i1}$ is the random effect parameter, which is different for every city. ${\varepsilon }_{ij}$ is the error term. The parameters of the random slopes model can be estimated by maximum likelihood estimation. The estimation was done using the lme4 package (version 1.1-31)^41^ in R-4.2.2.

### Ethical approval

This research did not require any ethical approval.

### Informed consent

This article does not contain any studies with human participants performed by any of the authors.

## Data Availability

The data that support the findings of this study are available from China Unicom but restrictions apply to the availability of these data, which were used under license for the current study, and so are not publicly available. Data are however available from the corresponding author upon reasonable request and with permission of China Unicom.
